# An overgrowth disorder associated with excessive production of cGMP due to a gain-of-function mutation of the natriuretic peptide receptor 2 (*NPR2*) gene

**DOI:** 10.1186/1687-9856-2013-S1-O43

**Published:** 2013-10-03

**Authors:** Kohji Miura, Noriyuki Namba, Keiko Yamamoto, Makoto Fujiwara, Yasuhisa Ohata, Taichi Kitaoka, Takuo Kubota, Toshimi Michigami, Keiichi Ozono

**Affiliations:** 1 Departments of Pediatrics, Osaka University Graduate School of Medicine, Japan; 2 Department of Bone and Mineral Research, Osaka Medical Center and Research Institute for Maternal and Child Health, Japan

## Aim

In human, overproduction of C-type natriuretic peptide (CNP) due to a chromosomal translocation was reported to cause skeletal dysplasia associated with tall stature. In addition, acromesomelic dysplasia, type Maroteaux, characterized by dwarfism is caused by loss-of-function mutations in the *Npr2* genethat encodes the CNP receptor NPR2.We report a three-generation family with tall stature, scoliosis and macrodactyly of the great toes, leading to a gain-of-function mutation in *Npr2*.

## Methods and results

Since the phenotype of the patients resembled several cases of the CNP overproduction phenotype, in terms of tall stature and large great toes, enhanced CNP/NPR2 signaling was suspected. Since the proband’s phenotype showed similarity to CATSHL syndrome, caused by a loss-of-function mutation in the *Fgfr3* gene, except for the absence of neurological symptoms, the *Fgfr3* gene was analyzed as well as the natriuretic peptide precursor C (*Nppc*), *Npr2*, and *Npr3* genesand a novel heterozygous G→A missense mutation at nucleotide +2647 (c.2647G→A) of the *Npr2* gene was identified.When expressed in HEK293A cells, the mutant *Npr2*cDNA generated intracellular cGMP in the absence of CNP ligand. In the presence of CNP, cGMP production was greater in cells that had been transfected with the mutant *Npr2*cDNA compared to wild-type cDNA. Transgenic mice in which the mutant *Npr2* was expressed in chondrocytes driven by the promoter and intronic enhancer of the *Col11a2* gene exhibited an enhanced production of cGMP in cartilage, leading to a similar phenotype to that observed in the patients.

## Conclusion

These results indicate that p.Val883Met is a constitutive active gain-of-function mutation and elevated levels of cGMP in growth plates lead to the elongation of long bones.Our findings reveal a critical role for NPR2 in skeletal growth in both humans and mice, and may provide a potential target for prevention and treatment of diseases caused by impaired production of cGMP, such as pulmonary hypertension, short stature, erectile dysfunction, heart failure, placental dysfunction, and dementia.

